# Targeting of Tetraspanin CD81 with Monoclonal Antibodies and Small Molecules to Combat Cancers and Viral Diseases

**DOI:** 10.3390/cancers15072186

**Published:** 2023-04-06

**Authors:** Christian Bailly, Xavier Thuru

**Affiliations:** 1 OncoWitan, Scientific Consulting Office, F-59290 Lille, France; 2 Institut de Chimie Pharmaceutique Albert Lespagnol (ICPAL), Faculty of Pharmacy, University of Lille, F-59006 Lille, France; 3 CNRS, Inserm, CHU Lille, UMR9020-U1277—Canther—Cancer Heterogeneity Plasticity and Resistance to Therapies, OncoLille Institut, University of Lille, F-59000 Lille, France

**Keywords:** CD81, cancer, drug targeting, natural products, therapeutic antibody, tetraspanins

## Abstract

**Simple Summary:**

Novel therapeutic targets are needed to improve treatments of aggressive cancers and viral diseases. Tetraspanins represent an emerging class of anticancer targets, notably the transmembrane protein CD81 which has been structurally well characterized. CD81 plays key roles in tumor growth and dissemination, and serves as a co-receptor for a number of viruses. The protein interacts with a variety of protein partners involved in different signaling pathways. Here, we provide an overview of the complementary approaches used to target CD81, with monoclonal antibodies and small molecules, including both natural products and synthetic compounds. Drug design approaches are discussed, as well as the limitations associated with the targeting of this ubiquitous protein. CD81 is considered as a promising anticancer and antiviral target.

**Abstract:**

Tetraspanin CD81 plays major roles in cell-cell interactions and the regulation of cellular trafficking. This cholesterol-embarking transmembrane protein is a co-receptor for several viruses, including HCV, HIV-1 and Chikungunya virus, which exploits the large extracellular loop EC2 for cell entry. CD81 is also an anticancer target implicated in cancer cell proliferation and mobility, and in tumor metastasis. CD81 signaling contributes to the development of solid tumors (notably colorectal, liver and gastric cancers) and has been implicated in the aggressivity of B-cell lymphomas. A variety of protein partners can interact with CD81, either to regulate attachment and uptake of viruses (HCV E2, claudin-1, IFIM1) or to contribute to tumor growth and dissemination (CD19, CD44, EWI-2). CD81-protein interactions can be modulated with molecules targeting the extracellular domain of CD81, investigated as antiviral and/or anticancer agents. Several monoclonal antibodies anti-CD81 have been developed, notably mAb 5A6 active against invasion and metastasis of triple-negative breast cancer cells. CD81-EC2 can also be targeted with natural products (trachelogenin and harzianoic acids A-B) and synthetic compounds (such as benzothiazole-quinoline derivatives). They are weak CD81 binders but offer templates for the design of new compounds targeting the open EC2 loop. There is no anti-CD81 compound in clinical development at present, but this structurally well-characterized tetraspanin warrants more substantial considerations as a drug target.

## 1. Introduction

Tetraspanins (TSPANs) are transmembrane proteins found in all cell types and tissues of eukaryotes [1]. They are involved in a large variety of biological processes including cell adhesion and mobility, cell signaling, protein trafficking, cell proliferation, neurotransmission, immune activation, reproduction and others [2]. These proteins, members of the transmembrane 4 superfamily, contribute importantly to cell-cell communication and trafficking of organelles. They are implicated in a large variety of human diseases, including viral and bacterial infections, inflammatory and metabolic diseases (hepatitis, diabetes,...) and cancers [3,4,5]. Over the past ten years, the implication of tetraspanins in cancer has been largely documented and several of these proteins are considered as valid targets for the design of novel anticancer agents. They are particularly adapted to the treatment of colorectal cancer, hepatocellular carcinoma, gastric cancer and other types of solid tumors [6,7,8,9]. These proteins are also considered in onco-hematology, notably for the treatment of acute myeloid leukemia (AML) and lymphoma [10]. For example, tetraspanin CD37 is expressed prominently on the surface of B cells and is chiefly implicated in the aggressivity of B-cell lymphoma [11,12]. Several monoclonal antibodies targeting CD37 are being developed for the treatment of B cell malignancies [13]. Globally, this emerging family of proteins attracts considerable attention for the design and development of small-molecules and bio-therapeutics for cancer and other human diseases [14].

There are over 33 tetraspanins in humans, plus non-conventional forms generated by alternative splicing [3,15]. Many of these tetraspanins are involved in cancers, chiefly TSPAN1, TSPAN8, TSPAN 13, CD9, CD37, CD63, CD81, CD82 and CD151 [6]. The latter protein CD151 can be targeted with monoclonal antibodies to block both primary tumor growth and metastasis in xenograft cancer models [16]. CD151 is considered a prime target to inhibit progression of prostate cancer [17] and could represent a valid target to combat triple-negative breast cancer [18]. CD151 can interact with another important member of the tetraspanin superfamily CD81 via its δ loop [19]. CD81 is a broadly expressed protein found on the plasma membrane of many cells, and often used as a marker for exosomes. This protein (previously known as TAPA-1, target of an antiproliferative antibody 1) has multiple cellular functions, notably as an important regulator of cell migration and invasion, and is now considered a key factor implicated in cancer progression [20]. Molecules selectively targeting CD81 could be of prime interest to treat different onco-malignancies, including B-cell and mantle cell lymphomas [21,22], acute lymphoblastic leukemia (ALL) [23] and perhaps AML. Indeed, it has been shown that CD81 represents an adverse prognostic marker in AML. The level of expression of this cell surface marker was associated with a worse prognosis in a cohort of 134 AML patients. CD81 expression had a negative impact on both overall survival and relapse-free survival [24].

The key roles played by CD81 in oncology and in other therapeutic areas, notably in the fields of viral infection and inflammation, has increased interest for the discovery of molecules, large and small, binding to CD81 and/or capable of neutralizing its effects. Tests and methods have been implemented to search for CD81-binding molecules and a few interacting compounds have been discovered and characterized. Taking profit of the known tridimensional structure of CD81, molecular models of small molecules bound to the protein have been elaborated and used as a guide to the chemical design of small molecule binders. The present review provides an updated analysis of the different CD81-binding compounds identified thus far and the perspectives to use these molecules as drug candidates and/or tools to help defining the biology of the protein and its implication in the complex tetraspanin network. After a brief analysis of the structure, dynamic organization and biology of CD81, the review focuses on the drug discovery and design aspects. The primary objective is to guide the identification of novel CD81-binding compounds, via a retrospective analysis of what has been carried out thus far, and a prospective view of tomorrow’s activities in this CD81 field. Our vision is essentially cancer-oriented, but the analysis shall serve to identify and design CD81 binders applicable in other therapeutic domains as well.

## 2. CD81 Structure and Dynamic Architecture

**Figure 1:**
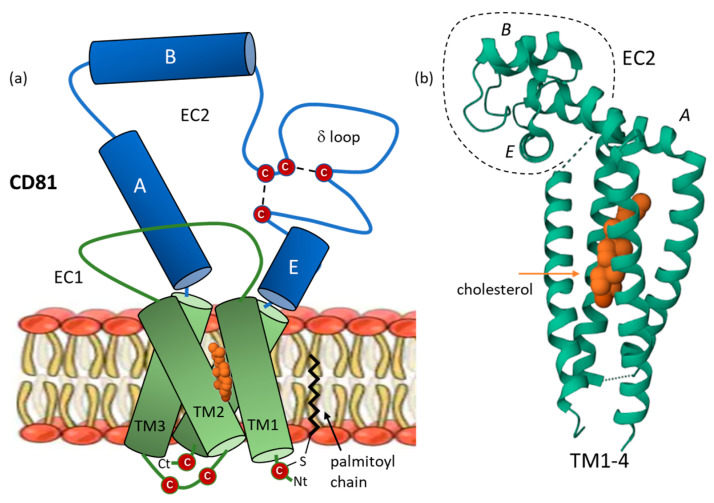
CD81 domain organization and structure. (**a**) Schematic representation of CD81 architecture, with four transmembrane (TM) segments embedded in the membrane bilayer and sequestering a molecule of cholesterol, and two extracellular (EC) loops. The small loop EC1 (or SEL) and the large loop EC2 (or SEL). The disulfur bridges between cysteine residue in EC2 are represented, as well as a palmitoylated cysteine residue contributing the anchoring of the protein into the membrane bilayer. (**b**) A model of CD81, with EC2 in a closed conformation (based in PDB: 5TCX).

Tetraspanins generally comprise four transmembrane domains and intracellular N and C termini. CD81 is a classical tetraspanin, with a standard transmembrane portion flanked by intra- and extra-cellular domains (Figure 1). The protein functions as a membrane receptor. It can be overexpressed in cultured cells and purified by immune-affinity [25]. At the structural level, the four transmembrane segments (TM1-TM4) of CD81 define a sort of funnel in which a molecule of cholesterol can bind. The funnel is represented by a four-stranded left-handed coiled coil. A large extracellular loop (EC2) caps the funnel, as a lid at the outer membrane leaflet (Figure 1). This large loop EC2 (or LEL) interacts with the vicinal smaller loop EC1 (or SEL) to modulate the conformation of the protein [26]. EC1 contains a small hydrophobic β-strand that packs in a conserved hydrophobic groove of the EC2, which itself is composed of two subdomains, including the δ loop [27,28]. The high-resolution crystal structure of CD81 has confirmed the presence of the two-disulfide bridge in the EC2 domain and a highly-conserved Cys-Cys-Gly motif. The purified full-length human protein has shown a monomeric form, whereas the crystal structure of the extracellular EC2 domain of human CD81 (88 of 236 residues) showed a dimeric assembly [29]. The dimeric form may serve to guide clustering of the different tetraspanin-binding proteins on the cell surface [30]. The organization of CD81 into domains is highly conserved among tetraspanins, notably the transmembrane domain structure, thus facilitating the realization of intra- and inter-molecular interactions and the assembly of the network into the so-called ‘tetraspanin web’ [28,31,32].

**Figure 2:**
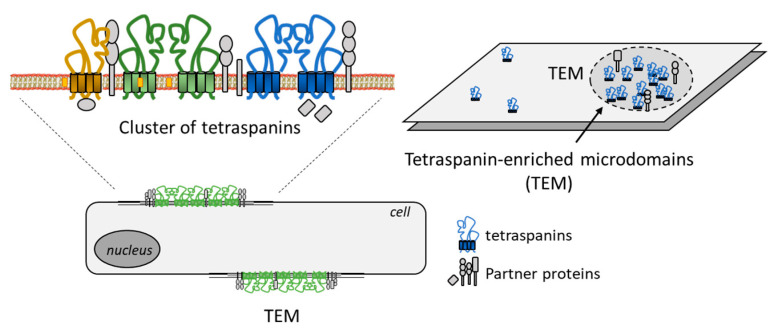
Representation of the clustering of tetraspanins to form microdomains at the plasma membrane, associating divers tetraspanins and partner proteins. The tetraspanin web is a dynamic structural organization at the cell surface, including promiscuous and specific interactions [32].

A specific feature of CD81 is its capacity to bind cholesterol. There is a well-defined cholesterol-binding pocket which is important to tune the conformation of the protein and exploited by certain viruses to enter into cells. CD81 serves as co-receptor for many viruses, including the hepatitis C virus (HCV), human immunodeficiency virus type 1 (HIV-1), herpes simplex virus 1 (HSV-1), influenza A virus (IAV), Chikungunya virus and a few others [4,33,34,35,36], and occasionally for certain bacteria, such as *Listeria monocytogenes* [37,38], and tropical parasites, such as *Plasmodium yoelii* [39] (Figure 2). The role of CD81 in HCV infection has been largely investigated. A potential allosteric mechanism by which cholesterol binding regulates the conformation of CD81 has been identified recently. The cholesterol-free open form of CD81 exhibits a reduced HCV receptor activity compared to the cholesterol-bound closed conformation which presents an enhanced activity for HCV entry. A conformational switch between the two forms operates, delimiting thus a CD81 cholesterol-sensing mechanism [40]. In its closed conformation, the CD81 extracellular loop EC2 disengages from EC1 and changes conformation. This process prevents the binding of CD81 with its main partner CD19 [41].

CD81 is a highly dynamic protein, subject to conformational changes which affect its receptor function and its activity in cells. The tetraspanin protein associates with different protein partners and with cholesterol in the membrane, so as to form protein clusters in membrane microdomains, designated tetraspanin webs or tetraspanin-enriched microdomains (Figure 2). These domains correspond to membrane area whereby tetraspanins organize functional higher-order protein complexes, upon interacting with each other and with other transmembrane proteins. CD81 can interact with itself and with other tetraspanins such as CD37, CD53 and CD82 to form individual clusters on the plasma membrane [42]. The sequestering of cholesterol molecules by CD81 within large molecular platforms of proteins induces local conformational changes that perturb the deformability of the membrane [43]. In addition, CD81 is subject to post-transcriptional modifications, notably to a palmitoylation within the intracellular N-terminal segment that is necessary to anchor the protein into lipid rafts [44]. The palmitoylation of cysteine residues of CD81 contributes to the association and anchorage of the protein into the microdomains [28,45]. In contrast, a protein ubiquitination contributes to the removal of the protein from the membrane, through clathrin-mediated endocytosis prior to lysosomal degradation of the protein [46].

## 3. CD81 Biology, Trafficking and Signaling

**Figure 3:**
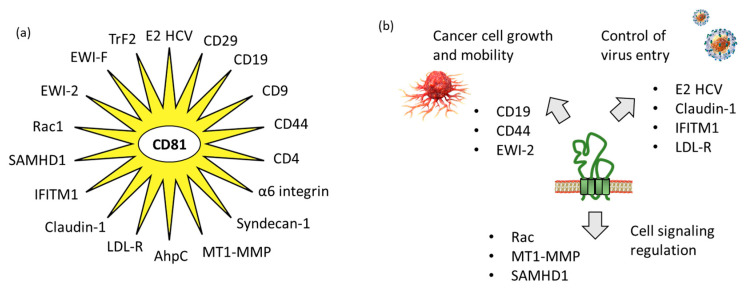
CD81 protein partners. (**a**) Multiple CD81-interacting proteins have been identified, including ten defined with the support of STRING database “https://cn.string-db.org/ (accessed on 1 March 2023)” and other additional proteins discovered through our own analysis of the scientific literature. (**b**) The partners include proteins interacting with the extracellular loops of CD81, such as proteins implicated in virus entry into cells and proteins involved in cancer cell growth and mobility, but also proteins interacting with the intracellular portions of CD81 for cell signaling.

Tetraspanins have multiple functions in cells. At the plasma membrane, they promote interactions with other membrane and intracellular proteins and lipids, to shape the organization of membrane domains. They are considered as “molecular facilitators” connecting extracellular and cytoplasmic signaling elements [47]. In this context, CD81 is known to facilitate cells adhesion or fusion in the frame of viral infection [48,49]. The large extracellular loop EC2 (LEL) of CD81 can interact with a variety of proteins, so as to facilitate cellular interaction and capture. We have identified 18 proteins which can interact directly with CD81 (Table 1). The tetraspanin certainly interacts with many other proteins, but true CD81-protein interactions have been evidenced experimentally in a limited number of cases. In other situations, interactions have been suggested based on a colocalization analysis using microscopy for example, but without definitive evidence of direct interaction between CD81 and its colocalization partner. Hereafter, we will limit our analysis to the various proteins for which a direct interaction with CD81 has been established (Figure 3).

### 3.1. CD81 Protein Partners Implicated in Virus Uptake

One of the prominent binders of CD81-LEL is the E2 envelope glycoprotein which plays a key role in the attachment and entry of the HCV virus into infected cells. CD81 increases the interaction of E2 with membranes and triggers a conformational change in E2 necessary for subsequent membrane fusion [50]. In fact, via E2 the virus utilizes different proteins as co-receptors, in particular, the tight-junction proteins claudin-1 and occludin together with CD81 and the protein SR-B1 (scavenger receptor class B member 1, encoded by gene *SCARB1*) [73]. SR-B1 is an 82-kDa transmembrane glycoprotein playing an important role in the regulation of cholesterol exchange between cells and high-density lipoproteins. Does CD81 directly interact with SR-B1? There is some evidence for that, notably data indicating that HCV E2 links a soluble form of CD81 and SR-B1 protein together. This physical neighboring could explain why both CD81 and SR-B1 are indispensable factors for HCV infection [74]. Moreover, the physical interaction between CD81 and claudin-1 has been firmly demonstrated. Claudin-1 oligomers associate with CD81 to form complexes playing a role in HCV infection [53,75]. In the same vein, a physical interaction has been evidenced between CD81 and protein IFITM1 (interferon-induced transmembrane 1) which is a hepatocyte tight junction protein implicated in HCV entry. The interaction of IFITM1 with HCV coreceptors CD81 and occludin disrupts the process of viral entry [54,55]. The low-density lipoprotein receptor (LDL-R) is involved also in viral entry. The formation of complexes between CD81, LDL-R and the serine proteinase PCSK9 (proprotein convertase subtilisin kexin type 9) has been observed [56]. PCSK9 enhances the degradation of the LDL-R and modulates liver CD81 levels [57]. However, the targeting of PCSK9 (implicated in cholesterol metabolism) with a mAb (alirocumab) does not affect CD81 and does not modulate HCV entry in hepatic cells [76].

### 3.2. CD81 Protein Partners Implicated in Tumor Growth and Dissemination

Another essential partner of CD81 is the B-lymphocyte coreceptor CD19, which is a key activator of the PI3K pathway and a prominent tumor-associated antigen. CD19 is a B/plasma cell-lineage marker and an essential target to combat B-cell malignancies, through the design of anti-CD19 chimeric antigen receptor (CAR) T-cell therapies [77]. For example, the CAR T-cell therapy axicabtagene ciloleucel has been recently approved for the treatment of relapsed or refractory follicular lymphoma [78]. Binding of CD19 to CD81 induces opening of the ectodomain and a reorganization of transmembrane helices, resulting in the occlusion of the cholesterol binding pocket [58]. This CD81-CD19 interaction, dynamically regulated upon B cell activation [79], is essential to the correct exposure of CD19 on the surface of B cells. Loss of CD81 expression results in an intracellular accumulation of CD19 [80]. Similarly, a mutation in the *CD81* gene leads to the expression of a truncated protein which does not enable CD19 maturation and cell surface expression [81]. The CD19-complex consisting of CD19, CD81, CD21 (also known as CR2, for complement receptor 2) and CD225 acts as a co-receptor to the B cell receptor (BCR). This complex cannot form properly when CD81 is mutated, causing severe diseases, but fortunately CD81 mutations are extremely rare in humans [82]. CD81 is an essential T cell costimulatory molecule. Its co-stimulation enhances naive T cell activation and largely modulates the activation of chimeric antigen receptors (CAR) [83].

CD81 associates with the two EWI proteins EWI-2 and EWI-F which are also partners of tetraspanin CD9 (Table 1). These two interactions are important because they have implications for cancer. EWI-2 (also called PGRL in mouse (prostaglandin regulatory-like protein)) is a regulator of both CD81 and CD9 functions [84], acting as a sequester to prevent the two tetraspanins from providing support to the TGF-β signaling. When EWI-2 binds CD9/CD81, the tetraspanins can no longer support the association of TGF-β receptors 1 and 2 (TβR2-TβR1). This signaling pathway is largely involved in melanoma growth, invasion and metastasis [59,85]. The suppression of CD81 with a shRNA in mesenchymal breast cancer has been shown to reduce primary tumor growth, extravasation and lung metastasis in vivo [86]. Therefore, the pharmacological blockade of CD81 could be an option to alter the malignant process.

The CD81/EWI-2 interaction has multiple functions, notably acting as a linker of the tetraspanin web to the actin cytoskeleton [87], and this interaction plays roles in different pathologies. CD81/EWI-2 interaction is a regulatory factor for glioblastoma cell growth and motility [60], but also for HCV and HIV infection [61,88]. Notably, the HIV-1 virus uses CD81-lined vesicle structures to infect astrocytes, and then these energy-consuming glial cells support trans-infection of HIV-1 to T-cells [89]. The virus uses CD81 as a rheostat to control different stages of the infection via interaction with different proteins such as EWI-2, but also the deoxynucleoside triphosphate phosphohydrolase SAMHD1 (sterile alpha motif and histidine aspartic acid domain containing protein 1) [68]. Through direct binding to SAMHD1, CD81 regulates the expression of the protein by promoting its proteasome-dependent degradation. This mechanism is implicated in the control of HIV-1 replication [68]. The viral restriction factor SAMHD1 is also considered as an anticancer target. The protein is frequently upregulated in cytarabine (Ara-C)-resistant AML [90]. SAMHD1 inhibitors are being developed for the sensitization of leukemia cells to nucleoside analogue-based therapy [91]. The link between SAMHD1 and CD81 may explain, at least in part, why CD81 is an adverse prognostic marker in AML [24].

The interaction of tetraspanins with the cell surface protein EWI-F (also known as CD9P-1 (CD9 Partner-1) or FPRP (F2alpha prostaglandin receptor regulatory protein)) has been well characterized. EWI-F is an immunoglobulin domain molecule which chiefly interacts with CD9 to form a 2:2 tetrameric arrangement implicated in the formation of tetraspanin-enriched microdomains [92]. In myoblasts, both CD9 or CD81 associate with EWI-F and the complex plays a role in muscle architecture (fusion of myotubes) and muscle regeneration [62]. Here again, the CD9P-1/tetraspanin complex functions as a regulator of cell motility [93]. Interestingly, a truncated form of EWI-F/CD9P-1, designated GS-168AT2, produced in human endothelial cells has been shown to inhibit angiogenesis and cell migration. GS-168AT2 corresponds to the sequence by which CD9P-1 physiologically associates with CD81 [94]. GS-168AT2 binds CD9 and CD81 and displays antitumor activity in vivo, associated with a downregulation of CD9 but not of CD81 [95]. This work suggested that the pharmacological modulation of the tetraspanin web could represent a new anti-angiogenic strategy [96]. The interaction between CD81 and CD9P-1 could also be exploited in the field of parasitic pathologies, because it has been shown that binding to and regulation of CD81 function by CD9P-1 represents a negative regulator of infection by *Plasmodium yoelii* (a malaria pathogen in rodent species) [39]. CD81 is implicated in the uptake of different pathogenic agents. A recent study highlighted the key role of CD81 in the uptake of pathogenic mycobacteria *Mycobacterium abscessus* through interaction with alkyl hydroperoxide reductase C (AhpC), a peroxiredoxin enzyme able to decompose several kinds of hydroperoxides [71]. The targeting of CD81 could be further exploited to combat different infectious diseases.

The hyaluronan-binding protein CD44 (a hyaladherin) is also a binding partner for CD81. A complex formation between these two membrane proteins has been evidenced recently [63]. They interact with each other through their extracellular regions and this recognition facilitates the formation of a tumor cell cluster and lung metastasis of triple negative breast cancer (TNBC). This interaction confirms the key role of CD81 in the migration and invasion of TNBC cells previously suggested by a proteomic analysis [18]. The invasion of TNBC cell lines can be halted with the use of a specific anti-human CD81 antibody (5A6) [97].

The CD81 interactome includes also the small GTPase Rac and the interaction plays roles in cancer cell motility and exosome formation [98]. The interaction of Rac (Rac1) with the C-terminal cytoplasmic domain of CD81 regulates the GTPase activity [99,100] and the modulation of Rac1 GTPase functions represents a key anticancer mechanism [101]. This tetraspanin-dependent regulation of cell motility raises an opportunity to control cancer cell migration with C81-targeted drugs. In addition, the CD81-Rac interaction plays a regulatory role in the innate and adaptive immunity against bacterial infection [38]. The signaling activity could be blocked with small molecules targeting the intracellular part of CD81 but probably also via targeting the extracellular protein loop.

Finally, we can evoke the association of tetraspanins with various integrins to modulate their function. Both tetraspanins CD9 and CD81 are a potential partner of αV integrin, at least in the testicular tissue where they are implicated in sperm development [102,103,104]. These two tetraspanins were also shown to link the cell adhesion molecule JAM-A to αvβ5 integrin and thus to play a role in the regulation of cell motility [105]. CD81 forms a complex with αV/β1 and αV/β5 integrins [106]. Integrins bind to tetraspanins such as CD81 via interaction with the constant region of the EC2 domain [105]. There are other integrins capable of interacting with CD81, such as α3β1 integrin, CD29, and others [65,106,107].

Altogether, our biological network analysis points out 17 protein partners for CD81 (Table 1). Most of them interact via the extracellular domain- of the tetraspanin, but in some cases the interaction concerns the intracellular portion, as depicted in Figure 3. There are probably many other partners, associated with CD81 itself or with CD81-containing tetraspanin platforms. A proteomic analysis mapped 33 host protein interactions of CD81 in primary human liver and hepatoma cells, including for example protein CAPN5 (calpain-5) and ubiquitin ligase CBLB (Casitas B-lineage lymphoma proto-oncogene B), capable of forming a complex with CD81 and implicated in HCV entry [108]. CD81 presents a dynamic expression profile and the multiplicity of partners is not surprising for a protein implicated in intercellular communication. Therefore, therapeutic molecules interacting with CD81 could serve as interrupters or regulators of different pathways and cellular processes. The next section provides an overview of CD81-binding molecules, large and small.

## 4. Regulation of CD81 Expression or Functions with Antibodies and Small Molecules

Our analysis began with the role of CD81 in leukemogenesis and the possibility to use this tetraspanin as a drug target to combat myeloid or lymphoid leukemias. The expression of CD81 varies significantly according to the leukemia type. CD81 expression at the surface of chronic lymphocytic leukemia (CLL) cells is dim in most cases [109,110]. CD81 alone is not a reliable marker to quantitate CLL cells but it is often included in a combination of markers (including for examples CD43, CD200, ROR1 and CD81) to quantify the residual disease and to optimize the identification of the CLL pathology [111]. The situation is more favorable in acute lymphoblastic leukemia (ALL) with a clearly defined role for CD81. The tetraspanin is also weakly expressed in residual pre-B-ALL [112] but it has been shown to play a key role in the bone marrow homing of leukemia cells. The knockout (KO) of CD81 (using the CRISPR/Cas9 technology) was found to reduce cellular adhesion and to disrupt bone marrow homing. Similarly, the down-regulation of CD81 surface expression with a combination of the histone deacetylase inhibitor panobinostat and the DNA-hypomethylating agent azacytidine was found to promote ALL cell mobilization from bone marrow to peripheral blood, thereby increasing the response to chemotherapy in disseminated patient-derived xenograft models [23]. Different studies have established a role for CD81 in leukemia cell engraftment, notably as a regulator for the re-entry of hematopoietic stem cells to quiescence, via a process implicating the Akt pathway [113,114].

CD81 KO cell lines provide a convenient system to appreciate the role of tetraspanin in various situations, notably to study virus infection and cancer cell aggressivity. The KO of CD81 in a neuroblastoma cell line was shown to reduce infectivity of HSV1 and HCV [35,115]. In cancer, the knockout of CD81 was shown to reduce tumor formation and metastasis. This trend has been particularly well demonstrated using the osteosarcoma cell line 143B which strongly expresses CD81. In this case, the corresponding KO cell line transplanted in mice exhibited a significantly reduced tumor growth and lung metastasis compared to the control group [116]. Moreover, CD81 KO mice exhibit an impaired immune response due to the altered capacity of CD81 to regulate the secretion of interleukin-10 (IL-10) in T regulatory (Treg) cells [117,118].

Another approach to modulate CD81 expression/function consists of using small interfering RNA (siRNA) against CD81. There are many examples of recent experimental studies using si-CD81 to control HCV entry and replication [119], to investigate the role of CD81 in radioresistance [120], or to investigate the senescence mechanism [121], for example. We will not further develop this aspect, to maintain a focus on therapeutic perspectives with antibodies and small molecules directed against CD81. These therapeutic agents could be developed to treat viral infections, some (myco)bacterial infections and different cancers. We choose to discuss the products globally, with reference to the type of products (mAbs vs. small molecules), not to the pathology. Their application can be diverse, but at present anti-CD81 have been essentially considered for the treatment of cancer.

### 4.1. Anti-CD81 Antibodies

**Figure 4:**
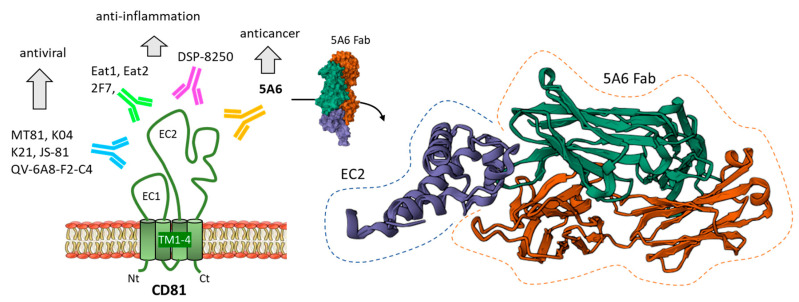
Different anti-CD81 antibodies (mAbs) have been discovered. They are used to study CD81 expression and/or function, notably to characterize membrane-bound CD81 on exosomes. Several anti-CD81 mAbs, such as QV-6A8-F2-C4, MT81, K04, K21 and JS-81 can be used to control virus entry into cells. The mAbs 2F7, Eat1 and Eat2 have been essentially used to study autoimmune encephalomyelitis and colitis, whereas mAb DSP-8250 has been tested against IBD. The lead mAb 5A6 displays marked antitumor activities, in murine models of B cell lymphoma and triple-negative breast cancer. A molecular model of the Fab fragment of 5A6 bound to CD81-EC2 is shown (PDB: 6U9S). 5A6 recognizes a conformational epitope on CD81 that is masked when CD81 is bound to CD19 [79].

The murine IgG1 antibody 5A6 (Figure 4) specifically targeting CD81 has been identified more than 30 years ago (designed by the late Prof. S. Levy, see acknowledgments) [122]. Early on, this mAb was used to demonstrate the role of CD81 in T cell activation, IL-2 production and proliferation [123]. Today, it remains one of the best tools commonly used to manipulate CD81. The therapeutic potential of this mAb has been investigated. Interestingly, 5A6 proved to be safe (not cytotoxic to normal PBMC) and equally efficient to rituximab (anti-CD20) to inhibit the growth of B cell lymphoma in a xenograft model. Humanized forms of the 5A6 mAb have been developed as candidates for the treatment of B cell lymphoma [124]. 5A6 recognizes a conformational epitope on the ectodomain of CD81, and this epitope is masked when CD19 is bound at the surface of B cells [79]. mAb 5A6 is very efficient at inhibiting the invasion of triple-negative breast cancer (TNBC) cells and the formation of metastases in xenograft models of TNBC [97]. 

Other anti-CD81 mAbs have been generated, such as the mAb DSP-8250 which recognizes a distinct epitope and presents an equally potent capacity to reduce T-cell migration, but no cytokine enhancement activity compared to 5A6 [125]. This mAb (from Dainippon Sumitomo Pharma, Osaka, Japan) has been tested for the treatment of intestinal bowel disease (IBD), but not developed further. There are other murine anti-CD81 mabs, such as the clones designated 2F7, Eat1 and Eat2, used to study experimental autoimmune encephalomyelitis and/or colitis. A long-lasting therapeutic effect on colitis in mice has been observed with 2F7 [126]. QV-6A8-F2-C4, MT81, K04, K21 and JS-81 are five other mAb targeting human CD81, used to block HCV E2 protein binding [127,128,129,130] (Figure 4). Among these mAbs, K21 is a therapeutic inhibitor of HCV entry, recognizing both human and monkey ECL2 CD81 with a high affinity (K_D_ = 0.4 nM) [131]. Many other neutralizing mAbs targeting CD81-EC2 have been raised to investigate CD81 functions, notably CD81-dependent virus entry into cells [132,133]. These research efforts have resulted in the design of optimized anti-CD81 with a high affinity for CD81-EC2 (sub-nanomolar K_D_) and a remarkable capacity to prevent HCV infection [134]. However, to our knowledge, there is no anti-CD81 in clinical development at present. In all cases, these mAbs are used for research purposes, and for example to isolate and characterize CD-81-lined exosomes by affinity chromatography [135]. CD81 is one of the most frequently used biomarkers for exosomes and other types of small extracellular vesicles [136,137].

### 4.2. CD81 Small Molecule Binders

The design of small molecules interacting selectively with CD81 is a challenge owing to the promiscuous nature of tetraspanins and the structural or functional redundancies between some tetraspanins. However, a selective targeting of a given tetraspanin is feasible considering their specific signatures in terms of dynamics, despite a common overall behavior [138,139]. The interaction of CD81 with partner proteins can be blocked with non-specific protein-protein interaction inhibitors, such as methylene blue [140]. However, the goal is to discover small molecules with a selectivity for CD81. One approach to obtain such molecules may be to start from the nonapeptide ATWVCGPCT which has been identified as a hCD81-like peptide, using a phage-displayed peptide library. This peptide is able to block the CD81 binding site of the HCV E2 protein [141]. Another peptide option is to use the motif SPQYWTGPA which is a mimotope of HCV E2 able to bind to hCD81 molecules [142]. A similar effect has been reported with the E2 peptide 710–725 which was shown to abrogate the interaction between CD81 and HCV E2 protein through the competitive binding of CD81 [143]. There are other peptides known to block the interaction between CD81 and the E2 protein of the virus [144]. Small-molecule-oriented phage display methods may be useful to generate anti-CD81 binders, although it is often a difficult process to transform peptides into small molecules without losing activity or selectivity [145].

**Figure 5:**
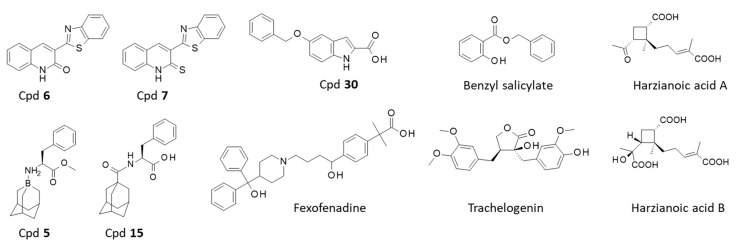
Small molecules identified as CD-81 binders. The binding of these molecules to the large extracellular loop (EC2) of CD-81 has been evidenced experimentally and/or proposed on the basis of computational studies (see Table 2).

**Figure 6:**
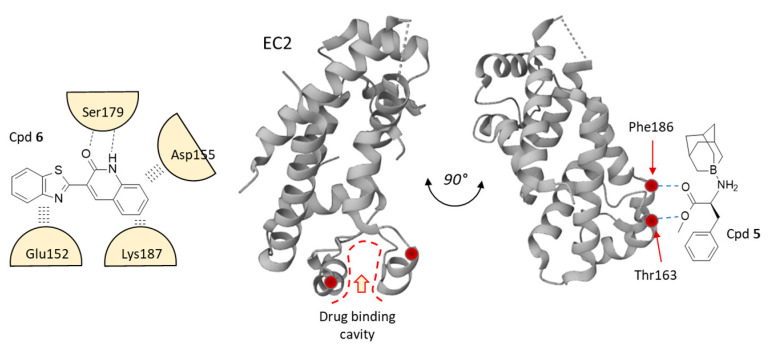
A representation of the drug binding cavity identified in the EC2 loop of CD81. The boraadamantane compound **5** and benzothiazole derivative **6** have been predicted to bind to CD81-EC2 on the basis of molecular docking and molecular dynamics simulation [146,148]. Specific interaction between the compound and the indicated amino-acid has been underlined. All compounds indicated in Figure 5 would fit into the binding cavity delineated by the indicated key residues.

There is a more direct way to search for CD81 binders (Table 2). Recently, Anand and coworkers have identified a series of quinoline derivatives able to bind and stabilize CD81 [146]. The lead compound in the series was the benzothiazole-quinoline derivative **6** (3-(benzothiazole-2-quinolinone) which has been predicted to bind to CD81, via interaction with residue Glu152, Asp155, Ser179 and Lys187 (Figure 5). The binding site for Cpd **6** is located at the extremity of the large extracellular loop (EC2-LEL) of CD81, overlapping with the HCV E2-binding surface (Figure 6). The binding site for Cpd **6** superimposes with the E2-binding site on CD81, a zone of about 806 Å^2^ which comprises residues Ile182, Phe186, Asn184 and Leu162 [147]. The replacement of the quinolinone moiety of **6** with a quinolinethione (Cpd **7**) afforded a compound with a slightly reduced predicted affinity for CD81 (binding affinity −6.9 vs. −6.3 kcal/mol) but the binding configuration remained essentially the same, according to the molecular docking and MD simulation [146]. This study opens perspectives to the design of CD81-targeted small molecules incorporating a benzothiazole-quinoline scaffold.

Another short series of CD81-binding small molecules has been identified several years ago. These compounds bear a 1-aminoadamantane core motif, such as compounds **5** (1-boraadamantane-L-phenylalanine methyl ester) and **15** ((*S*)-2-[(adamantane-1-carbonyl)amino]-3-phenylpropionic acid), represented in Figure 5 [148]. These molecules have been designed as inhibitors of HCV entry into cells, with a capacity to bind to CD81 and to block CD81-dependent proliferation of astrocytes, which significantly express CD81 on their membrane surface. The most active compound was Cpd **15** (Figure 5) which efficiently blocked astrocytes proliferation (82% inhibition at 5 µM), but presented a non-specific cytotoxic action at higher concentrations. Molecular models have been established with these compounds. Cpd **5** was shown to bind to the same cavity as the above-mentioned quinoline derivatives, establishing notably key H-bonds with residues Thr163 and Phe186 [148] (Figure 6). Cpd **15** can form stable complexes with CD81 EC2, stabilized by H-bonds and alkyl interactions. The models provide a useful guide to the design of analogues including the 1-aminoadamantane motif. However, it is worth underlining that at the clinical level, a study with the parent product amantadine has revealed no effect on CD81 expression when the product was administered to patients with chronic HCV infection, in contrast to a treatment with interferon-α which markedly down-regulated CD81 expression in those patients [154,155].

Other small molecules have been shown to block the interaction between CD81 EC2 and HCV, but they generally bind to the HCV E2 glycoprotein, to block binding to CD81 and thus inhibit HCV infection [156,157,158]. However, a few direct CD81 binders have been identified through a virtual screening approach, such as the indole derivative **30** (Figure 5) which was predicted to bind to CD81 EC2 and then experimentally confirmed as a ligand for CD81 using surface plasmon resonance (SPR). This 5-benzyloxyindole derivative is indeed a weak ligand for CD81 EC2 (K_D_ = 2.01 × 10^−4^ M) [149]. Two other synthetic molecules have been identified as CD81 binders: (i) the antihistaminic drug fexofenadine approved to treat seasonal allergic rhinitis and chronic idiopathic urticaria (a common skin allergy), and (ii) benzyl salicylate, a natural ingredient of many essential oils, largely used as a fragrance chemical (and an allergenic compound). Both compounds were identified as weak binders of CD81 EC2 by NMR spectroscopy [150]. Fexofenadine, an active non-toxic metabolite of terfenadine, was found to bind in a cleft-like region of the EC2 loop (via contacts with residues Leu154, Cys156, Val169, Ile180, Phe186 and Lys187) causing minimal structural perturbation upon binding. Its affinity was qualified as being weak (but not quantified) [150]. In a related study, the parent compound terfenadine has been shown to be a moderate inhibitor (27% inhibition at 50 µM) of the CD81-EC2 –HCV-E2 interaction [159]. In the benzyl salicylate series, analogues have been designed and tested but no better inhibitor than benzyl salicylate itself has been identified [160].

Interestingly, the natural product trachelogenin has also been shown to function as an inhibitor of HCV entry through CD81, possibly via a direct binding to the tetraspanin EC2 domain [151]. It is an antitumor dibenzylbutyrolactone-type lignan (Figure 5) isolated from diverse medicinal plants (*Trachelospermum asiaticum*, *Combretum fruticosum* and others) [161]. Trachelogenin binds to the same binding site as the quinoline derivatives cited above, via interaction with amino acid residues Thr166, Asn184, Lys187 and Glu188 on CD81 EC2. The mutation of residues Lys187 or Glu188 reduced the binding affinity [151]. However, CD81 is not the sole target of this natural product. Other protein targets have been proposed, notably central glutamate receptors [162]. The same mechanism of binding to CD81-EC2 extracellular domain and inhibition of HCV binding to CD81 has been invoked for the two products designated harzianoic acids A and B, isolated from the sponge-associated fungus *Trichoderma harzianum* (Figure 5). A direct binding to CD81-EC2 was evidenced using surface plasmon resonance (SPR) and the dissociation constant (K_D_) of 37.8 and 66.5 nM were determined with harzianoic acids A and B, respectively. This interaction may be at the origin of the anti-HCV activity of the two natural products, at least partially [152]. A few other natural products have been identified as potential CD81-binders (based on a molecular docking study), such as quercetin, puerarin and myricetin but they are flavonoids capable of binding to many proteins, generally with little or no selectivity [153].

Other options with small molecules could be considered to design CD81-binding compounds starting from molecules known to bind to other tetraspanins. For example, small molecules targeting the large extracellular loop (LEL) of tetraspanin CD151 have been identified recently. Pyrocatechol and the anticancer 5-fluorouracil (5-FU) have been characterized as CD151-LEL binders and inhibitors of CD151 expression [163]. In the same vein, a short cyclic peptide (cyclo-(L-leucyl-L-prolyl)) has been shown to bind CD151 and to block its interaction with the EGF receptor, leading to anticancer effects [164]. These are alternative options possibly transposable to target the LEL of CD81. It is also possible to regulate CD81 expression through an upstream targeting of the CD81 mRNA. This has been realized with the polyphenol epigallocatechin gallate (EGCG) which was found to enhance the expression of microRNA miR-548m directed against the 3’UTR region of CD81 mRNA. EGCG boosted miR-548m expression, thereby downregulating both CD81 protein and mRNA levels in HCV-infected Huh7 human hepatoma cells [165]. Similarly, EGCG has been shown to enhance expression of microRNA mir-194, leading also to a down-regulated CD81 expression [166].

The small molecules approach to targeting CD81 is attractive. It could offer a solution for the oral treatment of advanced solid cancers, notably to prevent metastasis. However, the challenge remains huge because the molecules discussed above are at an early stage of discovery, far from a clinical application. It is likely that the first development in this field will occur with a mAb, offering a much higher selectivity of action (but an injectable treatment), as is the case for other tetraspanins such as CD37.

## 5. Conclusions

The present analysis underlines the multiplicity of proteins partners for CD81 and the associated pleiotropic functions. The tetraspanin CD81 is a major modulator of virus entry into cells, chiefly studied in the context of HCV but also playing an important role for other pathogenic human viruses. CD81 is also implicated in the uptake of different pathogenic bacteria and mycobacteria [38,71]. At the same time, CD81 is an important regulator of cancer cell growth and mobility, now considered as a valid target to combat different types of solid tumors and lymphoid malignancies [8]. The role in myeloid malignancies is less clear, but it may also contribute to AML [24]. The role of CD81 and other tetraspanins in antitumor immunity reinforces the interest for the targeting of these proteins to combat cancer, notably virus-associated cancers such as gastric cancer [117,167]. No doubt, CD81 is a target of interest and CD81-interacting drugs could be exploited in various therapeutic domains, not limited to oncology and viral diseases.

On the other side, the pleiotropic roles of CD81 and the homology between different tetraspanins (for example CD9 and CD81) raise questions. As a pleiotropic protein, CD81 could be a profitable target to interrupt complex cellular processes, implicated in multigenic diseases such as cancers. Blocking CD81 could limit virus-entry, cancer cell proliferation and invasion, inflammatory processes and metastasis, for example. In this context, the antitumor effects observed with anti-CD81 mAbs 5A6 look promising. 5A6 proved to be safe in rodent models and it revealed marked anticancer activities in xenografted tumor models [88,115]. However, on the other side, a pleiotropic action may lead to more unwanted side effects and toxicities. The diverse biological actions of anti-CD81 mAbs will need to be finely dissected to identify the positive (pleiotropic) and negative (side effects) effects. The ubiquitous expression of CD81 in many tissues is a concern because it can increase the risk of side effects. In rodent models, the targeting of CD81 with mAbs has not caused major unwanted effects, but the development of molecules targeting CD81 will demand a careful assessment of the safety of the drug candidate to define the acceptable treatment dose, duration and potential dose-limiting toxicity events. Specific toxicity and toxicokinetic studies (notably in non-human primates) will be needed to define the efficacy vs. safety balance of an anti-CD81 therapy. There are options to limit potential systemic toxicity, if needed, such as local administration to tumor sites or the use of smart nanocarrier systems for small molecules in particular.

The potential redundancy between tetraspanins is perhaps not a major issue, because the similarities between tetraspanins are not so large and there are usually approaches to limit the target selectivity of small molecules (as is carried out with kinase inhibitors, for example). We have a lot to learn from the development of therapeutic monoclonal antibodies targeting tetraspanins which is now emerging. Anti-CD81 mAbs are not yet in the clinic but there are anti-tetraspanin mAbs in early clinical development, such as the anti-CD37 BI 836826 currently developed in leukemia and lymphoma [168,169]. Interesting data have also been reported with anti-CD9 mAbs or Fab in leukemia [170] and in colon cancer [171]. These data augur well for the potential development of anti-CD81, probably as a second-line therapy in combination with targeted therapy as is the case for BI 836826.

In the small molecule arena, the targeting of tetraspanin lags far behind the design and development of monoclonal antibodies. The situation is not unique, even common in the category of immune checkpoint inhibitors for example, with many mAbs approved (13 targeting the PD-1/PD-L1 checkpoint) but not a single small molecule in advanced clinical development against the same checkpoint, despite the many categories of anti-PD-L1 small molecules discovered [172,173,174]. However, in the tetraspanin field, the gap is huge because there is a major deficit of tetraspanin-binding small molecules. Only a handful of products capable of interacting with the EC2 loop of CD81 has been identified. Among the ten products listed (Figure 5), none can be qualified as a highly potent binder. The products, natural or synthetic, present a modest affinity for the EC2 loop, not extremely high and not always experimentally validated. The structurally complex natural products identified as CD81 binders, such as harzianoic acids A-B and trachelogenin may not be easy to convert into potent binders. The synthetic products, such as the benzothiazole-quinoline derivatives **6** and **7** will be easier to modulate as they are readily accessible by synthesis. The design of more potent CD81 binders can be guided by computational studies, making profit of the different tridimensional structures of CD81-EC2. The initial works have shown that it is possible to target tetraspanin with small molecules. Drug design approaches shall be encouraged to discover novel CD81-binders. With the advent of machine learning and optimization methods, the field of tetraspanin binders will hopefully see a rapid increase in developments and applications.

## Figures and Tables

**Table 1:** Proteins interacting with CD81.

Proteins	Types	Interaction and Effects	References
E2 HCV	Viral protein	The transmembrane E2 glycoprotein of HCV utilizes CD81 as a coreceptor for cell entry.	[50,51,52]
Claudin-1	Tight junction protein	The interaction CD81-Claudin1 contributes to HCV infection.	[53]
IFITM1	Tight junction protein	IFITM1 interacts with CD81 to limit HCV entry.	[54,55]
LDL-R	Membrane receptor	Interplay between CD81, LDL-R and PCSK9, to control HCV entry into hepatic cells.	[56,57]
CD19	Membrane receptor	CD81 and CD19 are core subunits of the B cell co-receptor complex. CD81 controls CD19 export activity, via a dynamically regulated process upon B cell activation.	[58]
EWI-2 (IgSF8, PGRL, CD316)	Signaling protein	The CD81/EWI-2 interaction contributes to the tetraspanin web and plays role in cancer cell growth and motility, and in HCV entry.	[59,60,61]
EWI-F (FPRP, CD9P-1)	Signaling protein	Complexes formed between EWI-F and CD81 (and CD9) play a role in the fusion of myotubes, which are essential elements of muscle architecture.	[62]
CD44	Adhesion molecule	The interaction between CD81 and CD44, through their extracellular regions, promotes tumor cell cluster formation and lung metastasis of triple negative breast cancer.	[63]
α6 integrin	Adhesion molecule	In male germ cells, CD81 interacts with α6 integrin subunit (which forms a dimer with β4 integrin). The complex plays a role in sperm maturation.	[64]
β1 integrin (CD29)	Adhesion molecule	Radiation was found to induce CD29/CD81 complex formation, thereby increasing the cellular uptake of exosomes.	[65]
Rac1	Small GTPase	Interaction of Rac with the C-terminal cytoplasmic portion of CD81 to regulate cell motility. Also has a role in bacterial infection.	[38]
CD4	Cell surface antigen	CD81 interacts with CD4 dimers concentrated in tetraspanin-enriched microdomains.	[66]
CD9	Tetraspanin	Tetraspanins CD9 and CD81 are involved in tetraspanin web formation in sperm. Molecular modelling suggests protein-protein interactions during sperm-egg membrane fusion.	[67]
SAMHD1	Enzyme	CD81 interacts with the deoxynucleoside triphosphate phosphohydrolase SAMHD1 and regulates its expression. The interaction promotes the proteasome-dependent degradation of SAMHD1. It is one of the metabolic regulators of HIV-1 replication.	[68]
MT1-MMP	Enzyme	Several tetraspanins, including CD81, associate with the membrane-type 1 matrix metalloproteinase (MT1-MMP) to regulate its cell surface localization and its function (notably its capacity to activate pro-MMP-2).	[69]
Syndecan-1	Proteoglycan	Knockdown of Syndecan-1 and CD81 inhibits HCV infection, suggesting their cooperative action. A direct interaction between the two proteins has been evidenced (using a proximity ligation assay).	[70]
AhpC	Enzyme	The mycobacterial enzyme alkyl hydroperoxide reductase C (AhpC) interacts with CD81-LEL to promote uptake of the pathogen by host cells.	[71]
TfR2	Membrane receptor	Transferrin receptor 2 (TfR2) is a binding partner for CD81. The interaction triggers RfR2 degradation by the ubiquitin E3 ligase GRAIL.	[72]

**Table 2:** Small molecules interacting with CD81.

Compounds ^1^ (Chemical Category)	CD81 Binding Information		References
	In Silico Data	Experimental Data	
Cpd **6**–**7** (synthetic products)	Docking and molecular dynamic simulation predict binding of the compounds to the EC2 of CD81.	None	[146]
Cpd **5**–**15** (synthetic products)	Molecular modeling suggests binding of the compounds to EC2 of CD81.	None	[148]
Indole derivative **30** (synthetic products)	Binding site on EC2-CD81 identified by molecular modeling.	Surface plasmon resonance (SPR) confirmed binding of Cpd **30** to CD81 EC2.	[149]
Benzyl salicylate Fexofenadine (synthetic products)	None	Weak binding to EC2-CD80 characterized by NMR spectroscopy.	[150]
Trachelogenin (natural product)	Molecular docking used to identify the contact points between trachelogenin and CD81 EC2.	The NP reduces interaction between HCV E2 and CD81. Direct binding to CD81 EC2 confirmed using protein mutants.	[151]
Harzianoic acids A-B (natural products)	A docking analysis supported binding of the two NP to CD81 EC2.	Direct binding to CD81-EC2 evidenced by SPR.	[152]
Flavonoids: quercetin, puerarin, myricetin. (natural products)	Molecular docking and molecular dynamic simulation predict binding of the flavonoids to CD81 EC2.	None	[153]

^1^ Compound structures are shown in Figure 5.

## Data Availability

Data sharing not applicable.
